# Supplementary material for: The midbody interactome reveals unexpected roles for PP1 phosphatases in cytokinesis
Source: Nat Commun. 2019 Oct 4;10:4513. doi: 10.1038/s41467-019-12507-9 (PMC6778137; doi:10.1038/s41467-019-12507-9)
Supplement: Supplementary file 1 — Supplementary Information [file 41467_2019_12507_MOESM1_ESM.pdf]

Supplementary Information for:

The midbody interactome reveals unexpected roles for PP1 phosphatases in cytokinesis

by

Capalbo et al.

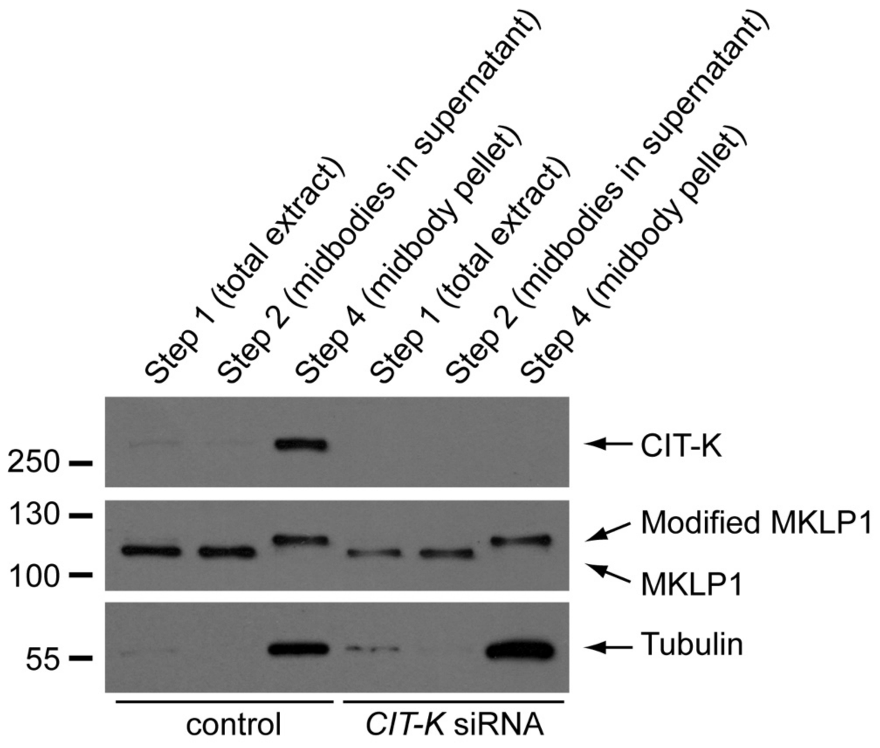

Supplementary Figure 1. Purified midbodies are enriched for known midbody proteins. HeLa cells treated with siRNAs directed against either a random sequence (control) or *CIT-K* were synchronized in telophase to purify midbodies and aliquots from the different purification steps (see Materials and Methods) were analyzed by Western blot to detect CIT-K, MKLP1 and tubulin. The numbers on the left indicate the sizes in kDa of the molecular mass marker. Note that the slower migrating band detected with the anti-MKLP1 antibody is very likely a post-translational modified form of MKLP1. Source data for Supplementary Figure S1 are provided as a Source Data file.

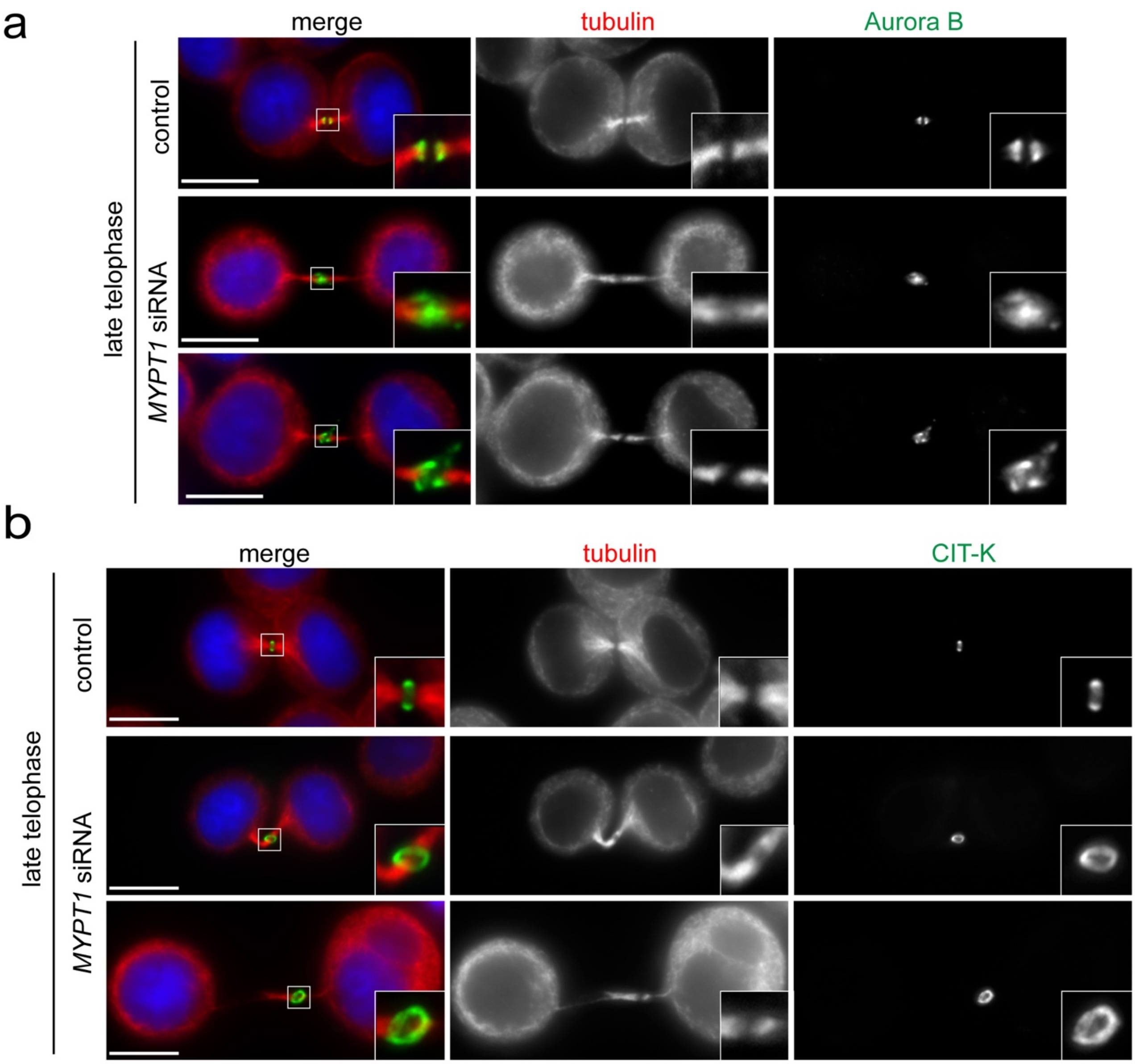

Supplementary Figure 2. Analysis of cytokinesis defects after MYPT1 depletion in HeLa S3 cells. (a-b) HeLa S3 cells were treated with siRNAs directed against either a random sequence (control) or *MYPT1* and after 48 hours were fixed and stained to detect DNA (blue in the merged panels), tubulin, and either Aurora B (a) or CIT-K (b). DNA condensation and the shape and thickness of microtubule bundles at the intercellular bridge were used as criteria to stage telophase cells. Insets show a 3x magnification of the midbody. Bars, 10 µm.

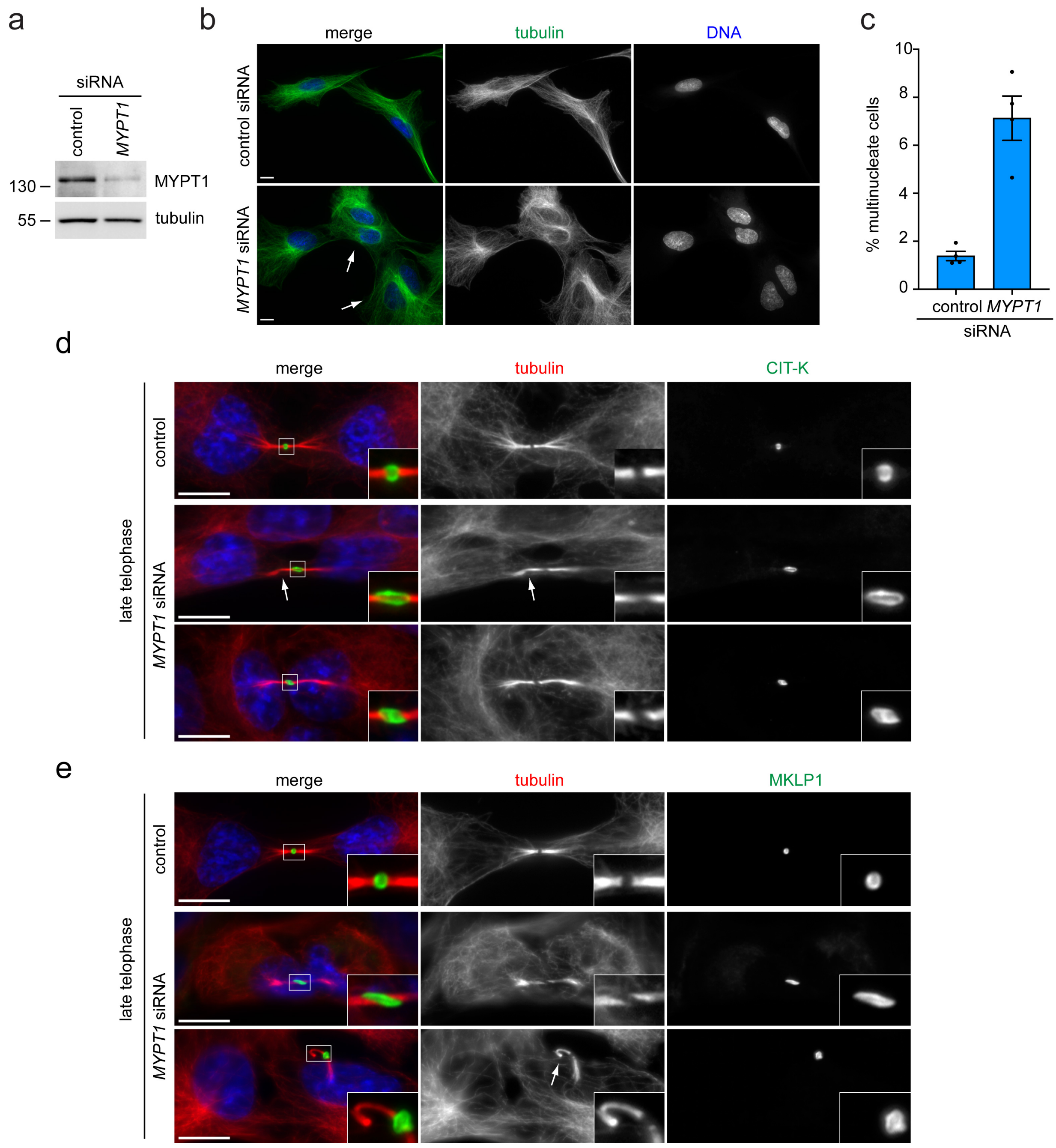

Supplementary Figure 3. Analysis of cytokinesis defects after MYPT1 depletion in RPE-1 cells. (a) RPE-1 cells were treated with siRNAs directed against either a random sequence (control) or MYPT1 and after 48 h proteins were extracted and analyzed by Western blot to detect the indicated proteins. The numbers on the left indicate the sizes in kDa of the molecular mass marker. (b) RPE-1 cells were treated with siRNAs directed against either a random sequence (control) or *MYPT1* and after 48 hours were fixed and stained to detect DNA and tubulin. The arrows indicate multinucleate cells. Bars, 10 µm. (c) Quantification of multinucleate cells obtained after control or *MYPT1* siRNA. More than 700 biologically independent cells were counted in n=4 independent experiments. Bars indicate standard errors. (d-e) RPE-1 cells were treated with control or *MYPT1* siRNAs and after 48 hours were fixed and stained to detect the indicated epitopes and DNA (blue in the merged panels). DNA condensation and the shape and thickness of microtubule bundles at the intercellular bridge were used as criteria to stage telophase cells. Insets show a 3x magnification of the midbody. The arrows mark a bent (d) and a broken (e) central spindle, phenotypes that are very similar to those observed in HeLa cells (Fig. 6). Bars, 10 µm. Source data for Supplementary Figure 3a and 3c are provided as a Source Data file.

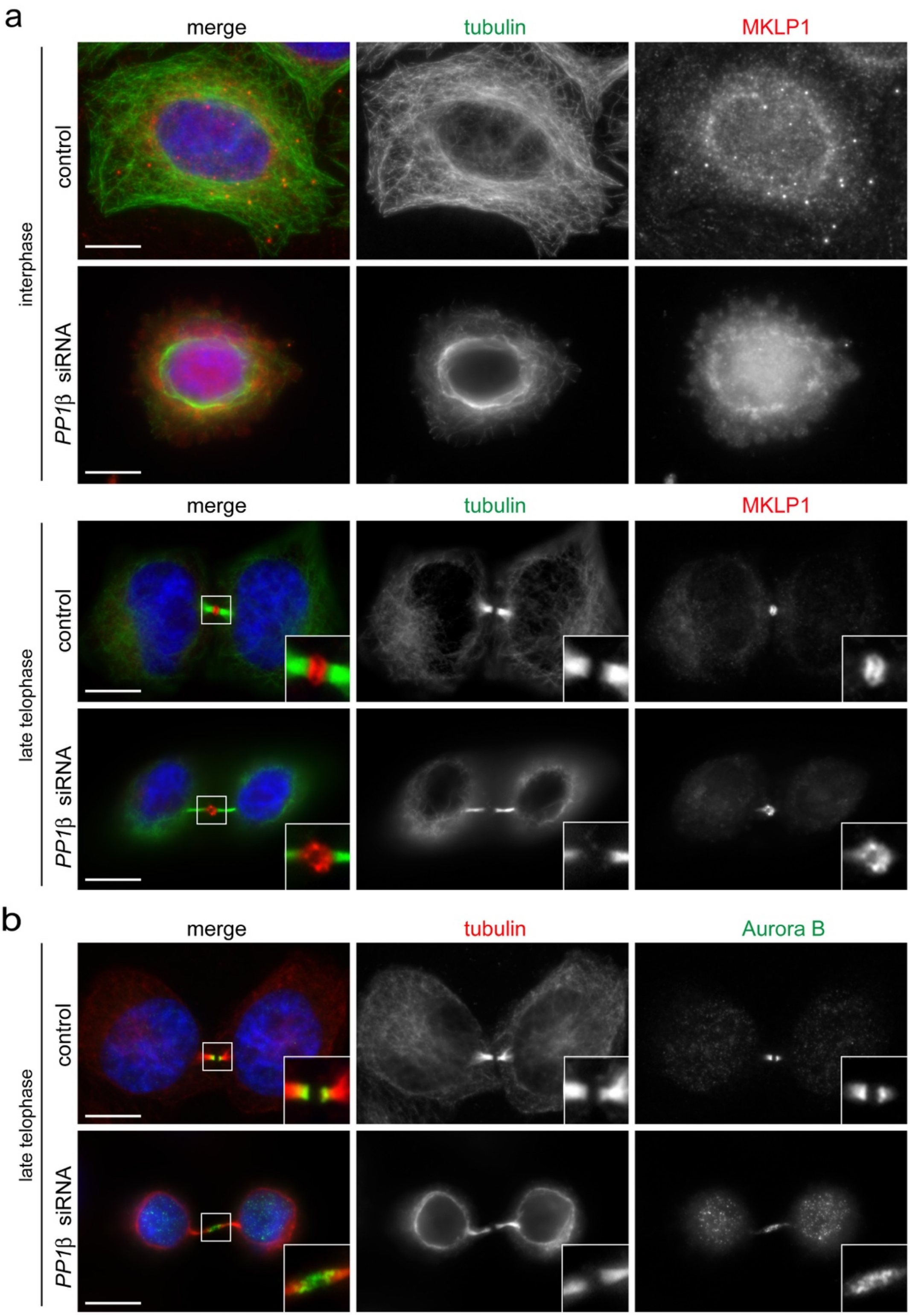

Supplementary Figure 4. *PP1β* siRNA phenocopies MYPT1 depletion. (a-b) HeLa Kyoto cells were treated with siRNAs directed against either a random sequence (control) or *PP1β* and after 48 hours were fixed and stained to detect DNA (blue in the merged panels), tubulin and either MKLP1 (a) or Aurora B (b). DNA condensation and the shape and thickness of microtubule bundles at the intercellular bridge were used as criteria to stage telophase cells. Insets show a 3x magnification of the midbody. Bars, 10 µm.

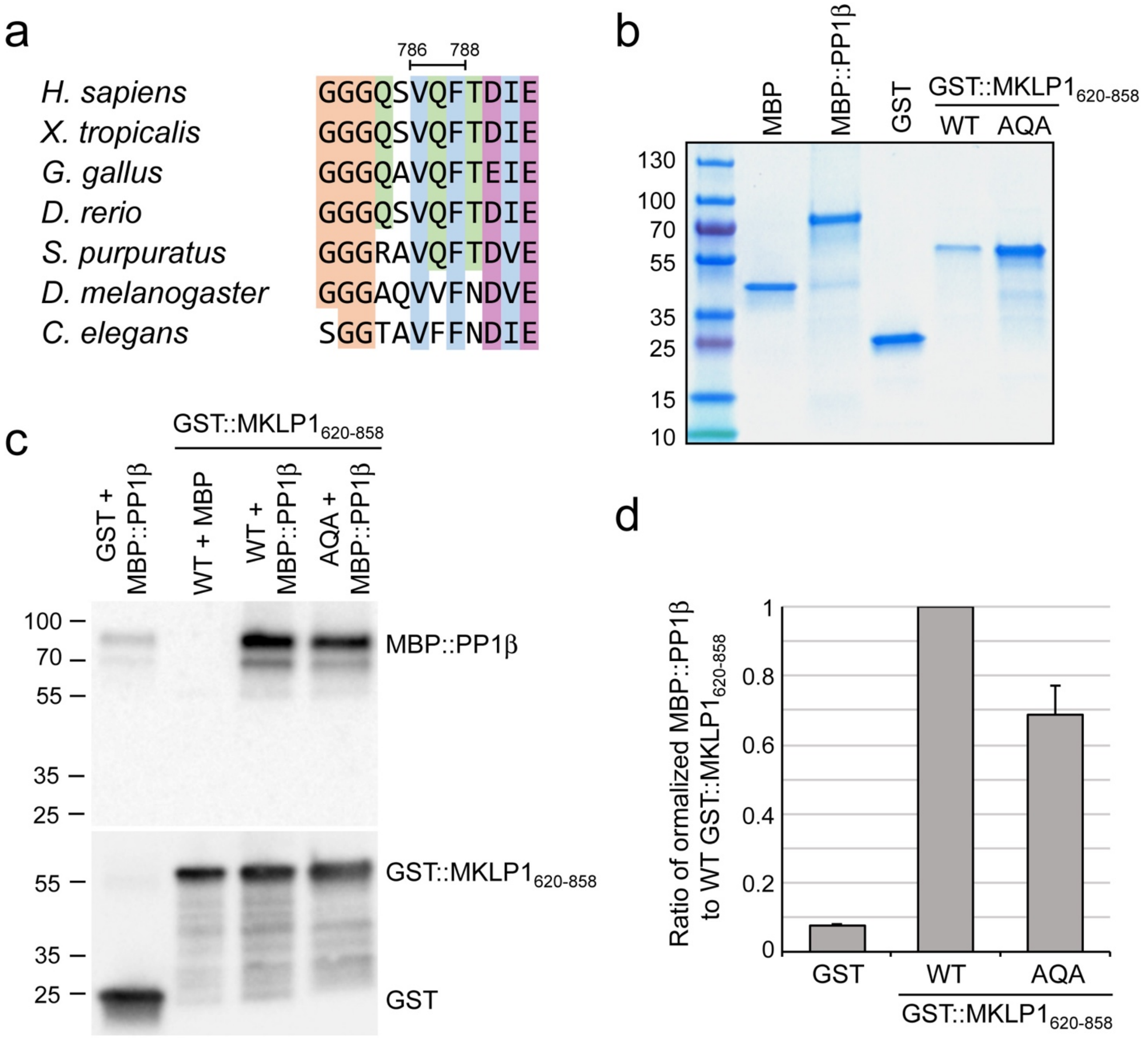

Supplementary Figure 5. PP1β interacts with a conserved binding site in the MKLP1 C-terminus. (a) The amino acid sequences containing the PP1β binding site (aa 786-788) of human MKLP1 and its orthologues in other vertebrate and invertebrate species were aligned using the Muscle algorithm[1]. Amino acids are colored according to their chemical properties. (b) Coomassie-stained gel showing the purified proteins used in the GST pull down assay. (c) GST-tagged wild type and AQA mutant MKLP1 fragments (aa 620-858) were expressed and purified in bacteria and then employed in a pull down assay with MBP**::** PP1β or MBP alone purified from yeast. Proteins were then analyzed by Western blot using antibodies against MBP (top) and GST (bottom). (d) Graph showing the quantification of the levels of MBP**::** PP1β normalized against their respective GST baits and the ratio of these values relative to WT GST:: MKLP1$_{620\text{-}858}$; n= 2 independent experiments. Bars indicate standard errors. Source data for Supplementary Figure 5c and 5d are provided as a Source Data file.

Supplementary Table 1. List of the proteins showing differential abundance at the midbody after CIT-K depletion.

Gene	Protein	log_2 ratio exp. 1	log_2 ratio exp. 2	*p*-value exp. 1	*p*-value exp. 2
CIT-K	Citron kinase	-2.69	-2.47	$2x10^{-45}$	$1.4x10^{-25}$
PC	Pyruvate carboxylase, mitochondrial	-0.80	-0.86	$3x10^{-9}$	$3.9x10^{-4}$
ASS1	Argininosuccinate synthase	-0.52	-0.63	$7.6x10^{-5}$	$5.5x10^{-4}$
FLNB	Filamin B	-0.50	-0.56	$1.2x10^{-4}$	$2.1x10^{-3}$
TIMM44	Mitochondrial import inner membrane translocase subunit TIM44	-0.40	-1.04	$1.4x10^{-3}$	$1.82x10^{-5}$
PPIB	Peptidyl-prolyl cis-trans isomerase B	-0.37	-0.57	$3.1x10^{-3}$	$1.8x10^{-3}$
AURKA	Aurora kinase A	0.45	0.69	$5x10^{-3}$	$2.6x10^{-4}$
CKAP2	Cytoskeleton-associated protein 2	0.45	0.67	$5x10^{-3}$	$3.7x10^{-4}$
PTPRF	Receptor-type tyrosine-protein phosphatase F	0.62	1.19	$2.3x10^{-3}$	$9.71x10^{-10}$
TPX2	Targeting protein for Xklp2	0.71	0.75	$4.3x10^{-6}$	$6.65x10^{-5}$
HNRNPH3	Heterogeneous nuclear ribonucleoprotein H3	0.83	0.97	$3.2x10^{-5}$	$5.47x10^{-7}$
KIFC1	Kinesin-like protein KIFC1	0.87	0.55	$1.5x10^{-5}$	$3.3x10^{-3}$

Proteins that were significantly less or more abundant (*p* value<0.01; significance B test corrected by Benjamini-Hochberg method) in CIT-K siRNA midbodies are listed. The corresponding normalized logarithmic ratios and corrected p-values from experiment 1 and experiment 2 are shown. Proteins shaded in blue were underrepresented (negative logarithmic ratios) after CIT-K depletion. Proteins shaded in red were overrepresented (positive logarithmic ratios).

Supplementary Table 2. List of the serine/threonine phosphatases present in the midbody interactome.

Baits	Anillin	Aurora B	CHMP4B	CHMP4C	CIT-K	ECT2	KIF14	KIF20A	KIF23/ MKLP1	PRC1	Proteome
**PPP1CA**	Score 183 Pept 5			Score 244 Pept 6	Score 1392 Pept 21	Score 400 Pept 9	Score 658 Pept 10	Score 303 Pept 8	Score 75 Pept 3	Score 1004 Pept 16	Yes
**PPP1CB**					Score 1312 Pept 19	Score 342 Pept 8	Score 85 Pept 1	Score 253 Pept 6		Score 963 Pept 14	No
**PPP1CC**	Score 267 Pept 9	Score 117 Pept 1	Score 302 Pept 6		Score 1355 Pept 20	Score 338 Pept 8	Score 690 Pept 12	Score 407 Pept 6	Score 68 Pept 2	Score 1067 Pept 19	Yes
**PPP1R9A**	Score 235 Pept 3		Score 120 Pept 2	Score 83 Pept 3		Score 254 Pept 4	Score 67 Pept 1	Score 48 Pept 1		Score 78 Pept 1	No
**PPP1R9B**	Score 237 Pept 5		Score 285 Pept 6			Score 340 Pept 7	Score 273 Pept 5	Score 94 Pept 1	Score 98 Pept 2		No
**PPP1R12A (MYPT1)**	Score 185 Pept 2		Score 197 Pept 3	Score 60 Pept 5	Score 572 Pept 6	Score 363 Pept 11	Score 855 Pept 15	Score 152 Pept 5		Score 564 Pept 13	Yes
**PPP1R12B**	Score 87 Pept 1						Score 83 Pept 1				No
**PPP1R12C**							Score 346 Pept 4	Score 87 Pept 1		Score 56 Pept 1	No
**PPP1R18**	Score 96 Pept 1		Score 128 Pept 1		Score 85 Pept 1	Score 232 Pept 4	Score 400 Pept 8	Score 201 Pept 7			No
**PPP1R13L**							Score 101 Pept 2	Score 222 Pept 2			Yes
**PPP2CA**				Score 38 Pept 1							No
**PPP2R5A**				Score 24 Pept 3	Score 107 Pept 2		Score 34 Pept 1			Score 98 Pept 2	Yes
**PPP2R1B**										Score 46 Pept 1	Yes
**PPP3CA**			Score 61 Pept 1			Score 63 Pept 1					No
**MPRIP**	Score 1358 Pept 23		Score 390 Pept 6	Score 43 Pept 3	Score 66 Pept 1	Score 179 Pept 6	Score 528 Pept 10	Score 432 Pept 5	Score 85 Pept 2		No
**CDC25A**		Score 48 Pept 9			Score 39 Pept 3					Score 35 Pept 1	No
**PGAM5**	Score 359 Pept 11		Score 75 Pept 2	Score 108 Pept 4	Score 61 Pept 4	Score 74 Pept 3	Score 430 Pept 13	Score 372 Pept 13	Score 171 Pept 6	Score 101 Pept 2	Yes

The baits are listed in the top row and the phosphatases in the far left column. Protein names are according to the UniProt database. Member of the PP1 family are shaded in green, while members of the PP2 family are shaded in red. The Mascot score (Score) and number of peptides (Pept) identified in each AP-MS experiment are indicated. The far-right column indicates whether the phosphatase was identified in the midbody proteome.

Supplementary Table 3. List of the oligonucleotides used in the experiments.

Name	Sequence 5'-3'	Purpose
PP1beta GST-pre yst for	TCCAAAATCGGATCTGGAAGTTCTGTTCCAGGGGC CCCTGATGGCGGACGGGGAGCTGAACGTGG	Plasmid to express GST-PP1β in yeast
PP1beta yst rev	CGGTTAGAGCGGATCTTAGCTAGCCGCGGTACCA AGCTTATCACCTTTTCTTCGGCGGATTAGCT	
MBPMKLP1 yst for	GAAAGACGCGCAGACTACGACCGAAAACCTGTAT TTTCAGTCCATGAAGTCAGCGAGAGCTAAGACAC	Plasmid to express MBP-MKLP1 in yeast
MKLP1 yst rev	CGGTTAGAGCGGATCTTAGCTAGCCGCGGTACCA AGCTTATCATGGCTTTTTGCGCTTGGGTTGT	
MBPPP1beta yst for	GAAAGACGCGCAGACTACGACCGAAAACCTGTAT TTTCAGTCCATGGCGGACGGGGAGCTGAACGTGG	Plasmid to express MBP-PP1β in yeast
MKLP1 AQA mutant primer	ATTAAGGGTGATATTTATAAAACAAGGGGTGGTG GACAATCTGCTCAGGCTACTGATATT	To mutate the VQF residues to AQA in MKLP1
PP1beta D94N for	CTATCTTTTCTTAGGAAATTATGTGGACAGAG	To generate the PP1β-dead mutant
PP1beta D94N rev	CTCTGTCCACATAATTTCCTAAGAAAAGATAG	
PP1beta H124N for	CTCTTAAGAGGAAACAATGAGTGTGCTAGCATC	
PP1beta H124N rev	GATGCTAGCACACTCATTGTTTCCTCTTAAGAG	

References

1 Edgar, R. C. MUSCLE: multiple sequence alignment with high accuracy and high throughput. *Nucleic Acids Res* **32**, 1792-1797, doi:10.1093/nar/gkh340 (2004).
