# Supplementary material for: The midbody interactome reveals unexpected roles for PP1 phosphatases in cytokinesis
Source: Nat Commun. 2019 Oct 4;10:4513. doi: 10.1038/s41467-019-12507-9 (PMC6778137; doi:10.1038/s41467-019-12507-9)
Supplement: Supplementary file 16 — Reporting Summary [file 41467_2019_12507_MOESM16_ESM.pdf]

nature research

Corresponding author(s): Pier Paolo D'Avino

Last updated by author(s): Sep 4, 2019

Reporting Summary

Nature Research wishes to improve the reproducibility of the work that we publish. This form provides structure for consistency and transparency in reporting. For further information on Nature Research policies, see Authors & Referees and the Editorial Policy Checklist.

Statistics

For all statistical analyses, confirm that the following items are present in the figure legend, table legend, main text, or Methods section.

n/a	Confirmed	
☐	☒	The exact sample size (n) for each experimental group/condition, given as a discrete number and unit of measurement
☐	☒	A statement on whether measurements were taken from distinct samples or whether the same sample was measured repeatedly
☐	☒	The statistical test(s) used AND whether they are one- or two-sided<br>*Only common tests should be described solely by name; describe more complex techniques in the Methods section.*
☒	☐	A description of all covariates tested
☒	☐	A description of any assumptions or corrections, such as tests of normality and adjustment for multiple comparisons
☐	☒	A full description of the statistical parameters including central tendency (e.g. means) or other basic estimates (e.g. regression coefficient) AND variation (e.g. standard deviation) or associated estimates of uncertainty (e.g. confidence intervals)
☒	☐	For null hypothesis testing, the test statistic (e.g. F, t, r) with confidence intervals, effect sizes, degrees of freedom and P value noted<br>*Give P values as exact values whenever suitable.*
☒	☐	For Bayesian analysis, information on the choice of priors and Markov chain Monte Carlo settings
☒	☐	For hierarchical and complex designs, identification of the appropriate level for tests and full reporting of outcomes
☒	☐	Estimates of effect sizes (e.g. Cohen's d, Pearson's r), indicating how they were calculated

Our web collection on statistics for biologists contains articles on many of the points above.

Software and code

Policy information about availability of computer code

Data collection	N/A
Data analysis	For protein identification and quantification in the SILAC experiments, the raw files were processed using MaxQuant (version 1.4.0.8) and the Andromeda search engine. For the identification of proteins from AP experiments, raw MS/MS data were analyzed using the MASCOT search engine (Matrix Science). AP-MS data were analyzed and visualized using Cytoscape (version 3.7.0). GO enrichment analysis was performed using PANTHER (version 14.0).

For manuscripts utilizing custom algorithms or software that are central to the research but not yet described in published literature, software must be made available to editors/reviewers. We strongly encourage code deposition in a community repository (e.g. GitHub). See the Nature Research guidelines for submitting code & software for further information.

Data

Policy information about availability of data

All manuscripts must include a data availability statement. This statement should provide the following information, where applicable:

- Accession codes, unique identifiers, or web links for publicly available datasets
- A list of figures that have associated raw data
- A description of any restrictions on data availability

All data are available in the main text or the supplementary materials. The SILAC mass spectrometry proteomics data have been deposited to the ProteomeXchange Consortium via the PRIDE partner repository with the dataset identifier PXD012374 (https://www.ebi.ac.uk/pride/archive/projects/PXD012374). The other proteomics data supporting the findings of this study are available within the paper and its supplementary information. All other data, materials and reagents are available from the corresponding author upon reasonable request.

Field-specific reporting

Please select the one below that is the best fit for your research. If you are not sure, read the appropriate sections before making your selection.

☒ Life sciences ☐ Behavioural & social sciences ☐ Ecological, evolutionary & environmental sciences

For a reference copy of the document with all sections, see nature.com/documents/nr-reporting-summary-flat.pdf

Life sciences study design

All studies must disclose on these points even when the disclosure is negative.

Sample size	N/A
Data exclusions	No data were excluded.
Replication	All experiments were replicated at least twice, as described in the manuscript.
Randomization	N/A
Blinding	Phenotypes in siRNA experiments were scored blindly by two different experimenters.

Reporting for specific materials, systems and methods

We require information from authors about some types of materials, experimental systems and methods used in many studies. Here, indicate whether each material, system or method listed is relevant to your study. If you are not sure if a list item applies to your research, read the appropriate section before selecting a response.

Materials & experimental systems

n/a	Involved in the study	
☐	☒	Antibodies
☐	☒	Eukaryotic cell lines
☒	☐	Palaeontology
☒	☐	Animals and other organisms
☒	☐	Human research participants
☒	☐	Clinical data

Methods

n/a	Involved in the study	
☒	☐	ChIP-seq
☒	☐	Flow cytometry
☒	☐	MRI-based neuroimaging

Antibodies

Antibodies used	The following antibodies were used in this study: mouse monoclonal anti alpha-tubulin (clone DM1A, Sigma, T9026), rabbit polyclonal anti beta-tubulin (Abcam, ab6046),), mouse monoclonal anti-cyclin B1 (clone GNS1, Santa Cruz, sc-245), mouse monoclonal anti-PP1 alpha (clone G-4, Santa Cruz, sc-271762), mouse monoclonal anti-PP1 beta (clone A-6, Santa Cruz, sc-365678), mouse monoclonal anti-PP1 gamma (clone A-4, Santa Cruz, sc-515943), mouse monoclonal anti-CIT-K (BD Transduction Laboratories, 611377), mouse monoclonal anti-MYPT1 (clone C-6, Santa Cruz, sc-514261), (Abcam, ab2254), rabbit polyclonal anti-MKLP1 (clone N19, Santa Cruz Biotechnology, sc-867), rabbit polyclonal anti-phospho MKLP1 pS70828, rabbit polyclonal anti-tri-phospho CHMP4C pS210 pS214 pS21526, mouse monoclonal anti-Aurora B (clone AIM-1, BD Transduction Laboratories, 611082), mouse monoclonal anti-PRC1 (clone C-1, Santa Cruz, sc-376983), rabbit monoclonal anti-phospho PRC1 pT481 (Abcam, ab62366), rabbit polyclonal anti-phospho-histone H3 pS10 (Merck, 06-570), rabbit polyclonal anti-mono-phospho MRLC pS19 (Cell Signaling Technology, 3671), rabbit polyclonal anti-di-phospho MRLC pT18 pS19 (Cell Signaling Technology, 3674), goat polyclonal anti-RacGAP1 (Abcam, ab2270), mouse monoclonal anti-GST (Abcam, ab92), mouse monoclonal anti-MBP (NEB, E8032).
Validation	The antibodies directed against PP1 alpha, PP1 beta, PP1 gamma and MYPT1 were validated by means of siRNA-mediated depletion followed by Western blot and immuno-fluoresence analyses (Fig. 4). The antibody against PRC1 was validated by confirming that its distribution was identical to that of the GFP-tagged transgene used for the AP-MS experiments (Table 1) and to that of other previously published studies (e.g. Hu et al. Mol Biol Cell 23, 1024-1034, 2012). All other antibodies have been validated either in this work or in our previous published studies.

Eukaryotic cell lines

Policy information about cell lines

Cell line source(s)	The sources of all cell lines used in this study are listed in the manuscript text and in Table1.

Authentication	Cell lines were not authenticated.
Mycoplasma contamination	All the cell lines used in this study were tested for mycoplasma contamination and found to be negative.
Commonly misidentified lines<br>(See ICLAC register)	N/A
